# Polarization-Modulated Optical Homodyne for Time-of-Flight Imaging with Standard CMOS Sensors

**DOI:** 10.3390/s25061886

**Published:** 2025-03-18

**Authors:** Ayaka Ebisu, Takahito Aoto, Tsuyoshi Takatani

**Affiliations:** 1 Deggree Programs in Systems and Information Engineering, University of Tsukuba, Tsukuba 305-8573, Japan; 2 Optech Innovation, LLC., Tsukuba 305-0031, Japan; aoto@optechinnovation.com; 3 Institute of Systems and Information Engineering, University of Tsukuba, Tsukuba 305-8573, Japan

**Keywords:** depth imaging, spatial resolution, optical correlation, photoelastic modulator

## Abstract

Indirect time-of-flight (iToF) imaging is a widely applied technique to obtain a depth image from the phase difference of amplitude-modulated signals between emitted light and reflected light. The phase difference is computed via electrical correlation on a conventional iToF sensor. However, iToF sensors face a trade-off between spatial resolution and light collection efficiency because it is hard to downsize the circuit of the electrical correlation in a pixel. Thus, we propose a novel iToF depth imaging system based on polarization-modulated optical homodyne detection with a standard CMOS sensor. A resonant photoelastic modulator is employed to modulate the polarization state, enabling optical correlation through interaction with an analyzer. The homodyne detection enhances noise resistance and sensitivity in the phase difference estimation. Furthermore, the use of a polarization camera allows to reduce the number of measurements. We first validate the successful estimation of the phase difference in both setups with an avalanche photodiode or a CMOS sensor. The experimental results show accurate depth estimation even in challenging factors such as a low signal-to-noise ratio, temporal intensity variations, and speckle noise. The proposed system enables high-resolution iToF depth imaging using readily available image sensors.

## 1. Introduction

Depth imaging has emerged as a critical technology across numerous applications, including autonomous driving, factory automation, and virtual/augmented reality. Time-of-flight (ToF) imaging represents a widely adopted approach for obtaining depth information due to its practical advantages of low cost and accessibility. By leveraging the constant speed of light, ToF systems can estimate scene distances by measuring the time interval between light emission and detection after scene interactions.

There are two primary ToF methodologies: direct ToF (dToF) and indirect ToF (iToF). dToF techniques require high-speed, precise light measurement, typically implemented through a combination of avalanche photodiodes (APDs) and time-to-digital converters. However, integrating these components into dense arrays presents significant technical challenges. Consequently, iToF approaches are generally preferred for applications demanding high spatial resolution, as they can more readily achieve dense pixel arrays while maintaining acceptable depth measurement capabilities.

**Figure 1:**
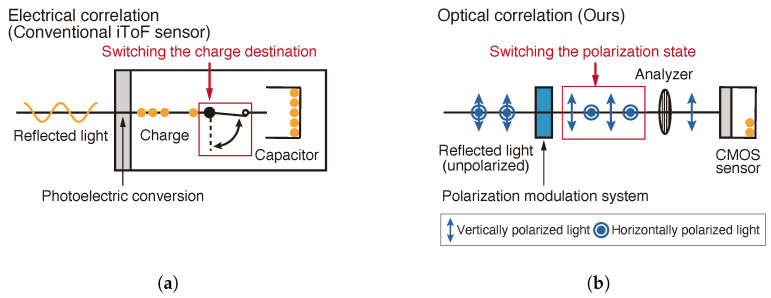
Comparison of correlation operations between the conventional and proposed approaches. (**a**) Electrical correlation operation in conventional iToF sensors. (**b**) Optical correlation operation using polarization modulation.

iToF imaging systems [1,2,3] illuminate targets with intensity-modulated light and estimate flight time by measuring the phase difference between emitted and reflected light signals through temporal correlation operations. Each pixel in an iToF image sensor integrates both a light-sensing element and temporal correlation circuitry (Figure 1a), enabling independent depth measurements at each spatial location. However, this integrated architecture imposes fundamental constraints on the achievable spatial resolution of iToF cameras. While a higher resolution requires reducing the pixel size within fixed sensor dimensions, the temporal correlation circuitry occupies a minimum area that cannot be readily scaled down. Consequently, increasing the spatial resolution necessitates reducing the light-sensing area of each pixel, creating an inherent trade-off between spatial resolution and light collection efficiency.

Two main approaches have been developed to address this spatial resolution limitation. The first leverages compressive sensing to achieve super-resolution depth imaging that exceeds the native capabilities of iToF sensor arrays [4,5,6]. This method utilizes a digital mirror device (DMD) with higher spatial resolution than the iToF sensor to modulate the reflected light. By applying time-varying spatial modulation patterns, a high-resolution depth image corresponding to the resolution of the DMD can be reconstructed from multiple measurements. These methods use spatial encoding with digital mirror devices and diffraction gratings to restore high-resolution depth images by increasing sparsity. However, this method generally yields only a slight improvement in spatial resolution, and only by a factor of several.

The second approach utilizes standard high-resolution CMOS image sensors. In this approach, the phase difference is not calculated on the time correlation circuit on the image sensor, but rather by optical correlation operations. The synthetic wavelength imaging method and the fast optical shutter method are two methods of optical correlation calculation. The former method uses two light sources with close wavelengths (the frequency of the light itself) and measures the phase shift of the beat wave resulting from their optical interference, enabling high-resolution depth measurement [7,8,9]. Although the frequency of the light is very high (THz), the phase shift can be measured even with standard CMOS sensors by using low-frequency beat waves. However, these techniques require two highly coherent lasers, which are expensive and require large pieces of equipment.

In contrast, the latter method uses a high-speed optical shutter (Figure 1b) to perform the correlation computation by charge distribution that has been performed in conventional iToF cameras (Figure 1a). As high-speed optical shutters, image intensifiers [10,11], electro-optic modulators (EOMs) [12], and electro-absorption modulators (EAMs) [13,14,15,16] are used. Image intensifiers require high voltages of several kV and are not suitable for implementation in general consumer-use cameras. EOMs may also require voltages as high as several several hundred volts to several kV (e.g., car cells and Pockels cells), but in combination with resonant circuits, they can be driven at low voltages (several tens of volts) at certain frequencies. They can be modulated at high frequencies in the MHz to GHz range, but the bulkiness of the material crystals and the small photosensitive area mean that depth images must be acquired by scanning. EAMs are also capable of MHz modulation at low voltages and they don’t require scanning to obtain a depth image. They are made of wafers containing multiple quantum wells, which are more complex structures than the single crystal wafers used in EOMs.

In addition to these methods, Atalar et al. [17,18,19,20,21,22] have constructed a polarization modulation-based optical shutter with a resonant photoelastic modulator for iToF imaging. This technique uses heterodyne detection, in which slightly different frequencies are set for light source modulation and polarization modulation, the resulting beat signal is captured by the camera at the differential frequency, and waveform characteristics (phase, amplitude, and offset) are estimated from the time variation of brightness. On the other hand, there is a method of detection called homodyne detection, also known as lock-in detection, that uses a modulated signal and a demodulated signal of the same frequency. Homodyne detection typically demonstrates superior noise resistance compared to heterodyne detection’s waveform-based approach. This enhanced robustness leads to improved sensitivity and accuracy in the estimation of phase difference. Indeed, Atalar [23] acknowledges the potential advantages of incorporating homodyne techniques in future implementations.

In this paper, we propose a novel system of polarization-modulated optical homodyne for iToF imaging. The proposed system mainly consists of a resonant photoelastic modulator and a standard CMOS image sensor. By operating the resonant photoelastic modulator, the angle of linear polarization is temporally modulated, enabling optical correlation through interaction with an analyzer. This configuration allows depth measurement with high spatial resolution using a CMOS image sensor. In this work, we apply a homodyne approach using the identical frequency for both the light source modulation and demodulation signals towards phase difference detection with high sensitivity and precision. Furthermore, we propose a method using a polarization image sensor as a light receiving element, because the use of polarization modulation enables phase shift setting of the demodulated signal depending on the angle of the detector. This method has the advantage of reducing the number of measurements for the optial homodyne method. Due to the relationship between the modulation of the linear polarization angle and the detector angle, four phase-shifted images can be obtained with two exposures by using four polarization angle detectors of a polarization image sensor.

Table 1 presents a comparison of different methods. The proposed method enhances the number of pixels in ToF cameras by capturing the results of optical correlation using a standard CMOS sensor. Compared to conventional iToF sensors, utilizing a high-pixel-count CMOS sensor addresses the issue of spatial resolution. Additionally, while the heterodyne method requires reconstructing phase information from the beat wave based on the intensity variations of multiple images, the proposed method employs the homodyne approach, allowing phase difference estimation from just four phase-shifted images. Furthermore, since the proposed method performs demodulation using an optical shutter based on polarization modulation, a polarization camera with analyzers at different angles enables reducing the number of measurements from four to one. This is not feasible with optical shutters based on light amplification or absorption modulators.

## 2. Polarization-Modulated Optical Homodyne

### 2.1. Principle of Polarization Modulation

Polarization modulation refers to the temporal modulation of linear polarization angles using devices capable of electrically controlling polarization states. Specifically, a polarization modulator consists of a phase modulation element that can delay the slow axis phase according to the applied voltage, combined with a quarter-wave plate (QWP). In this paper, we perform polarization modulation using a phase modulation element based on the inverse piezoelectric effect and photoelastic effect. When an electrical signal is input to this optical phase element, strain is generated in the element due to the inverse piezoelectric effect, and the phase delay of the slow axis varies through the photoelastic effect according to the magnitude of this strain.

**Figure 2:**
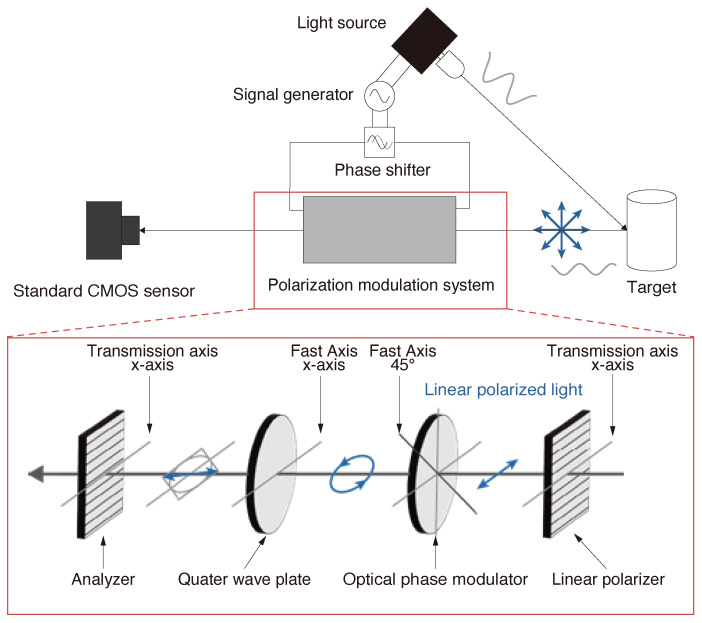
The concept of our method and polarization modulation system.The blue arrows represent the polarization states.

Figure 2 shows the mechanism of the linear polarization light change by the polarization modulator. Firstly, we assumed that the light reflected on a target object surface is unpolarized. The reflected light passes through a linear polarizer so that its transmission axis is horizontal (x-axis) and the horizontal component is only transmitted. Then, the linearly polarized light passes through the OPM that is arranged so that its fast axis is 45° to the *x*-axis and changes into any elliptically polarized light depending on the applied voltage. By passing the elliptically polarized light through a QWP so that its fast axis direction is aligned with the original linearly polarized light, the elliptically polarized light returns to linearly polarized light with the different angle.

### 2.2. Optical Transmission Through Polarization Modulation

**Figure 3:**
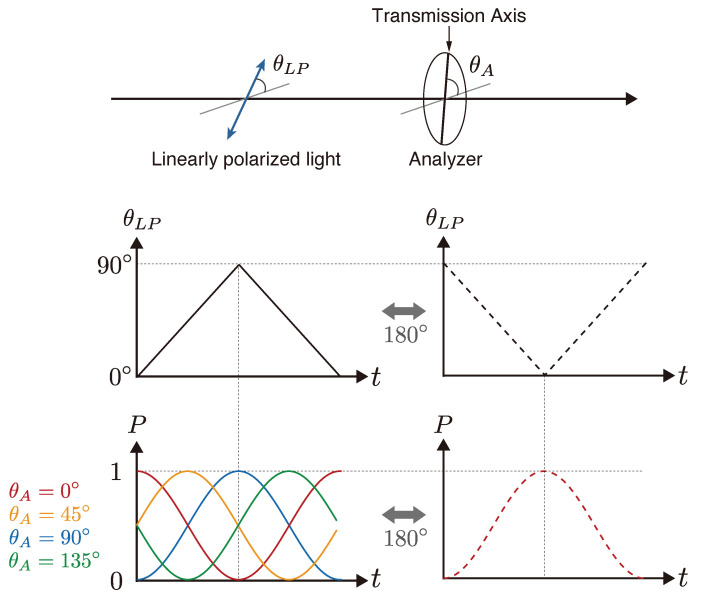
The relationship between optical transmission and both the linear polarization angle and analyzer angle.

When a linear polarizer (analyzer) is placed after the polarization modulator, the optical transmission varies according to the angle of the linear polarization passing through the analyzer. Figure 3 illustrates the relationship between optical transmission and both the linear polarization angle and analyzer angle. Consider $\theta_{LP}\left(t\right)$ as the time-varying angle of linear polarization due to polarization modulation, where $\theta_{LP}\left(t\right)$ varies linearly from 0° to 90° during the first half-period and from 90° to 0° during the second half-period. When an analyzer with a transmission axis angle $\theta_{A}$ of 0° is positioned in this configuration, the optical transmission P(t) follows the pattern shown in Figure 3 (red line). A phase shift of 180° in $\theta_{LP}\left(t\right)$ results in a corresponding 180° phase shift in optical transmission (red dashed line). Since the phase of $\theta_{LP}\left(t\right)$ is determined by the input signal phase to the optical phase modulator, the phase can be controlled electronically.

Furthermore, when the phase of $\theta_{LP}\left(t\right)$ is reset to 0° and the analyzer angle $\theta_{A}$ is set to 90°, the optical transmission exhibits a phase inversion (180° phase shift, blue line) compared to when $\theta_{A}$ is 0°. When $\theta_{A}$ is set to 45° and 135°, the optical transmission phases δ become 90° (yellow line) and 270° (green line), respectively. Thus, the optical transmission phase can also be controlled through the analyzer angle, where the phase shift in optical transmission is twice the analyzer angle ($\delta =2\theta_{A}$). Since the polarization image sensor is equipped with analyzers at four polarization angles ($\theta_{A}=0{}^{\circ},\;45{}^{\circ},\;90{}^{\circ},$ and 135°), intensities at four phase shifts for the four-bucket method can be theoretically acquired in a single exposure. However, in practice, multiple exposures may be required due to the limited magnitude of polarization modulation, as discussed in Section 5.2.

### 2.3. iToF Depth Measurement Using Polarization-Modulated Optical Homodyne

In this subsection, we describe the method for iToF depth measurement using polarization modulation. In our method, a target object is irradiated with intensity-modulated light in the same way as conventional iToF methods. The irradiated light L(t) is expressed by the following equation:

(1) $$L\left(t\right)=A_{L}\cos\left(2\pi f_{m}t\right)+B_{L},$$

where *t* is time, $A_{L}$ is the amplitude of the irradiated light, $f_{m}$ is the modulation frequency, and $B_{L}$ is the offset of the irradiated light.

The irradiated light is reflected at a certain point on the object surface and returns to the camera. The reflected light R(t) is expressed by the following equation:

(2) $$R\left(t\right)=A_{R}\cos\left(2\pi f_{m}t+\phi \right)+B_{R},$$

where $A_{R}$ is the amplitude of the reflected light, ϕ is the phase difference between the irradiated light and the reflected light, and $B_{R}$ is the offset of the reflected light.

The proposed method differs from conventional methods in that it uses optical transmittance control by polarization modulation to calculate the phase difference. Polarization modulation is changing the angle of linear polarization light temporally and the light transmittance is changed along with the angle between the direction of linearly polarized light and the transmission axis of the analyzer. The light transmittance P(t) is, thus, given by

(3) $$P\left(t\right)=\alpha \cos\left(2\pi f_{d}t+\delta \right)+\beta ,$$

where α is the amplitude of the light transmittance, $f_{d}$ is the demodulation frequency, δ is the phase shift between the irradiated light and the light transmittance, and β is the offset of the light transmittance. $f_{d}=f_{m}$ because our proposed method is based on optical homodyne technique. Ideally, polarization modulation that the angle of the linear polarization light repeats increases and decreases in the range from 0° to 90°. In that case, α,β=0.5 and the transmittance changes in the range from 0 to 1. The intensity of the reflected light observed by each pixel is described as follows:

(4) $$\begin{array}{cc} I\left(\delta \right) & =\lim_{n\to \infty }\frac{1}{n}\int_{0}^{nT}R\left(t\right)P\left(t,\delta \right)dt \end{array}$$

(5) $$\begin{array}{cc} \; & =A_{I}\cos\left(\phi -\delta \right)+B_{I}, \end{array}$$

where *n* is the natural number, *T* is the modulation period, $A_{I}=\frac{A_{R}\alpha T}{2}$, and $B_{I}=B_{R}\beta T$.

The phase difference ϕ between incident and reflected light can be calculated by the four-bucket method [1,24] as in the conventional iToF methods. In our method, the phase is calculated using four measurements obtained by varying the optical transmission phase at 0°, 90°, 180°, and 270°. The equations for the intensities at these four phase shifts and the phase difference calculation are shown below.

(6) $$\begin{array}{cc} I_{0} & =I\left(0\right)=A_{I}\cos\left(\phi \right)+B_{I}, \end{array}$$

(7) $$\begin{array}{cc} I_{90} & =I\left(\frac{\pi }{2}\right)=-A_{I}\sin\left(\phi \right)+B_{I}, \end{array}$$

(8) $$\begin{array}{cc} I_{180} & =I\left(\pi \right)=-A_{I}\cos\left(\phi \right)+B_{I}, \end{array}$$

(9) $$\begin{array}{cc} I_{270} & =I\left(\frac{\pi }{2}\right)=A_{I}\sin\left(\phi \right)+B_{I}, \end{array}$$

(10) $$\begin{array}{cc} \phi & =\arctan\left(\frac{I_{270}-I_{90}}{I_{0}-I_{180}}\right)\;. \end{array}$$

The amplitude $A_{I}$ and offset $B_{I}$ of the observed intensity can also be calculated from the above four observations as follows:

(11) $$\begin{array}{c} A_{I}=\frac{\sqrt{{\left(I_{0}-I_{180}\right)}^{2}+{\left(I_{270}-I_{90}\right)}^{2}}}{2}, \end{array}$$

(12) $$\begin{array}{c} B_{I}=\frac{I_{0}+I_{90}+I_{180}+I_{270}}{4}\;. \end{array}$$

Using the calculated phase difference and the light speed *c*, the depth from camera to the target *d* is calculated by

(13) $$d=c\frac{\phi }{4\pi f_{m}}\;.$$

## 3. Implementation

### 3.1. Fabrication of Optical Phase Modulator

**Figure 4:**
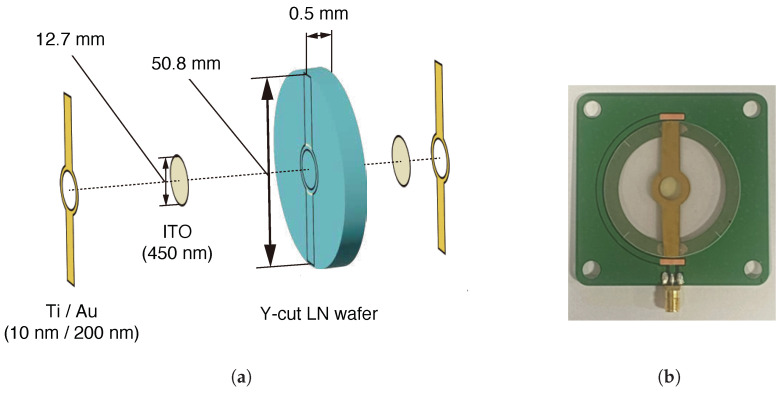
Fabrication of optical phase modulator. (**a**) Schematic diagram of the device structure. (**b**) Photograph of the fabricated modulator.

In this paper, we fabricated a optical phase modulator based on the photo-elastic effect caused by the mechanical resonance characteristics of piezoelectric material, with reference to [19]. Figure 4 shows the schematic of the optical phase modulator (a) and its photograph (b). The substrate of the optical phase modulator is a lithium niobate (LN) wafer (Y-cut) that has piezoelectric properties. A 450 nm film of indium tin oxide (ITO) was deposited on both sides of the wafer in a circular area with a radius of 6.35 mm from the center of it. The ITO films function as a transparent conductive film. Also, 10 nm and 200 nm of titanium and gold were deposited as electrodes on the ITO films, respectively. The film shapes of these materials are shown in Figure 4a and were deposited by sputtering with metal masks. The film-formed LN wafer was connected to a circuit board using conductive paste and aluminum wire to drive the optical phase modulator by an external signal.

### 3.2. Modulation Frequency

Since the optical phase modulator is based on the mechanical resonance characteristics of piezoelectric materials, the magnitude of the angular change in the final output linearly polarized light varies depending on the driving frequency of the optical phase modulator. In addition, as mentioned in Section 2.1, the amplitude of the light transmittance changes depending on the magnitude of the angular change in the linearly polarized light of the polarization modulator, and it affects the intensity of the reflected light observed by the camera. Since the brightness of the reflected light observed by the camera is an important factor related to the accuracy of phase estimation, it is desirable to drive the polarization modulator at a frequency that increases the amplitude of the light transmittance due to polarization modulation. Therefore, in order to determine the frequency used to drive the optical phase modulator, we conducted an experiment to examine the amplitude of the light transmittance at each drive frequency.

**Figure 5:**
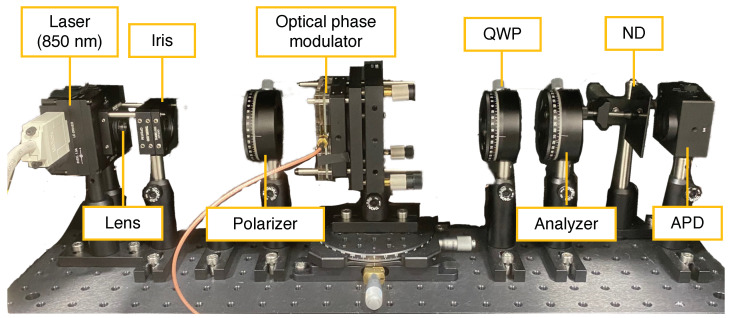
Through-beam-type experimental setup with an avalanche photodiode (APD).

Figure 5 shows the experimental setup. We use a laser at 850 nm wavelength (L850P200, Thorlabs, Inc., Newton, NJ, USA) and an avalanche photodiode (APD) (APD430A/M, Thorlabs, Inc., Newton, NJ, USA) as a single-pixel camera. Between the laser and the APD, a linear polarizer (WGPF-30C, SIGMAKOKI Co., Ltd., Tokyo, Japan), the optical phase modulator, a quater wavelength plate (QWP) (WPQW-NIR-4M, SIGMAKOKI Co., Ltd., Tokyo, Japan), and an analyzer (WGPF-30C, SIGMAKOKI Co., Ltd., Tokyo, Japan) are placed. The modulation signals for the light source and the optical phase modulator are generated using two synchronized function generators (DG4000, RIGOL TECHNOLOGIES, Co., SuZhou, China). Since the output of the function generator was insufficient to drive the optical phase modulator, we used a motor driver (MTO-EV021, Marutsuelec Co., Ltd., Tokyo, Japan) to amplify the modulation signal to the extent that polarization modulation could be achieved.

In this experiment, only polarization modulation was performed without modulating the light source, and changes in brightness due to the polarization modulator were observed using APD. The signal detected by APD was captured via a spectrum analyzer (Rigol DSA815). By frequency analysis of the observed signal using a spectrum analyzer, the signal level at the driving frequency was extracted, and the frequency at which the signal level was high was determined by a sparse-density search.

**Figure 6:**
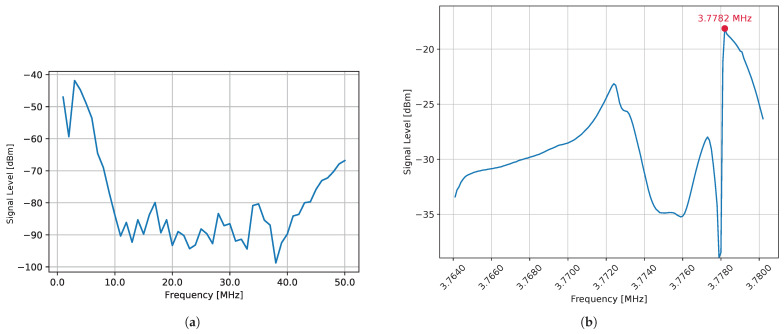
Results of search for driving frequency. (**a**) Measurement at 1 MHz intervals over the range of 1 MHz to 50MHz. (**b**) Measurement at 100 Hz intervals over the range of 3.7641 MHz to 3.7802 MHz. The peak frequency was 3.7782 MHz.

The results are shown in Figure 6. The horizontal axis represents the driving frequency, and the vertical axis represents the signal level. Figure 6a presents measurement results obtained at 1 MHz intervals over a frequency range from 1 MHz to 50 MHz. The highest signal intensity was observed around 3 MHz, indicating that this range includes frequencies where brightness variations due to polarization modulation are maximized. Based on this, a finer search was performed around 3 MHz. As shown in Figure 6b, measurements taken at 100 Hz intervals from 3.7641 MHz to 3.7802 MHz revealed that the signal intensity peaked at 3.7782 MHz. This suggests that when the modulator operates at this frequency, resonance occurs, leading to the maximum polarization modulation amplitude. In contrast, despite differing by only 200 Hz, the signal intensity at 3.7780 MHz dropped significantly, likely due to anti-resonance. Since a 20 dBm decrease in signal intensity results in a 90% reduction in amplitude, precise frequency tuning is crucial. Therefore, 3.7782 MHz was chosen as the driving frequency for the experiment.

### 3.3. The Angle of Analyzer

As described in Section 2.3, if the range of angular variation of linear polarization due to polarization modulation is from 0° to 90°, the range of variation of light transmittance is from 0 to 1. However, when the fabricated phase modulator was driven, it was found that the change in light transmittance was smaller than ideal. This is probably due to insufficient power supplied to the modulator. Since an RF amplifier with a frequency of 3.7 MHz and high output power was not available, a motor driver with a full-bridge switching circuit was used. Note that the output of the motor driver is a square wave and contains harmonics other than the modulation frequency.

When the angular variation range of linear polarization due to polarization modulation is less than 0 to 90 degrees, the analyzer angle becomes crucial for maximizing the amplitude of light transmission. Considering intensity variations of transmitted light with respect to analyzer angle for a given linearly polarized light, if the polarization angle change from the phase modulator remains constant, the modulation amplitude should be maximized when the analyzer is set at angles where the derivative of the intensity function with respect to analyzer angle is at its maximum or minimum. Conversely, if the analyzer angle is set to the angle at which the derivative of this intensity function is zero, the modulation amplitude will be minimal. In our method, the analyzer angle should be set so that the modulation amplitude is maximized.

**Figure 7:**
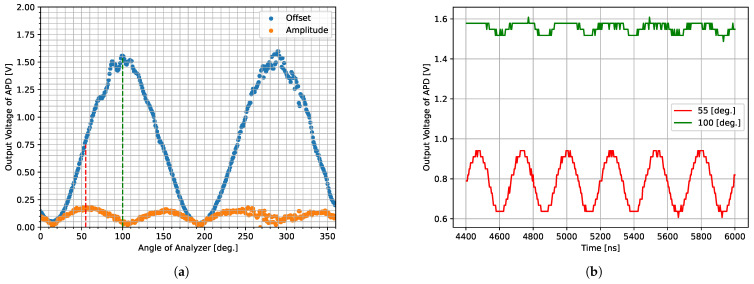
Variation in polarization modulation performance with analyzer angle. (**a**) Offset (blue) and amplitude (orange) of polarization-modulated light intensity versus analyzer angle. (**b**) Waveforms of polarization-modulated light intensity at specific analyzer angles: red shows the waveform at 55° where the offset is maximum, and green shows the waveform at 100° where the derivative of the offset is maximum.

To verify this hypothesis, we performed measurements using the same setup as in Section 3.2, driving only the polarization modulator without light source modulation. We rotated the analyzer and measured APD output signal waveforms at various analyzer angles using an oscilloscope (DS1104Z, RIGOL TECHNOLOGIES, Co., Suzhou, China). Figure 7a shows the offset and amplitude of the observed signals plotted against analyzer angle. The results demonstrate that amplitude becomes minimal at angles where the derivative of the offset is zero. Conversely, the amplitude increases at angles where the absolute value of the offset derivative is large. For example, Figure 7b shows waveforms at 100° (the absolute value of the offset derivative is maximum) and 55° (where the derivative of the offset is maximum). The amplitude at 55 is approximately five times larger than at 100°, with the offset also being about half as large, indicating superior modulation performance. Note that 55° is just one example, and that 145°, 235°, and 325° with the largest absolute derivative values are also acceptable. In subsequent sections, experiments are conducted at analyzer angles that provided higher modulation performance.

## 4. Validation

### 4.1. Phase Difference Measurement Using an APD

To validate the modulator’s capability for phase difference measurements via optical homodyne detection, we first employ an avalanche photodiode (APD) as the detector, leveraging its high temporal resolution and sensitivity. We utilize a through-beam configuration, as illustrated in Figure 5 in Section 3.2.

The light source amplitude modulation and polarization modulation are both driven at 3.7782 MHz. We systematically vary the phase difference between these two modulation signals from 0° to 360° in 1° increments. At each phase setting, we acquire data and estimate the corresponding phase difference. We capture the temporal intensity variations, which arise from the correlation between light source and polarization modulations, using a oscilloscope (DS1104ZPlus, RIGOL TECHNOLOGIES, Co., SuZhou, China). Phase difference estimations are calculated from waveform data accumulated over a fixed time period. The oscilloscope is configured to record 3000 measurement points with a sampling rate of 350 MSa/s and a time scale of 100 ns.

**Figure 8:**
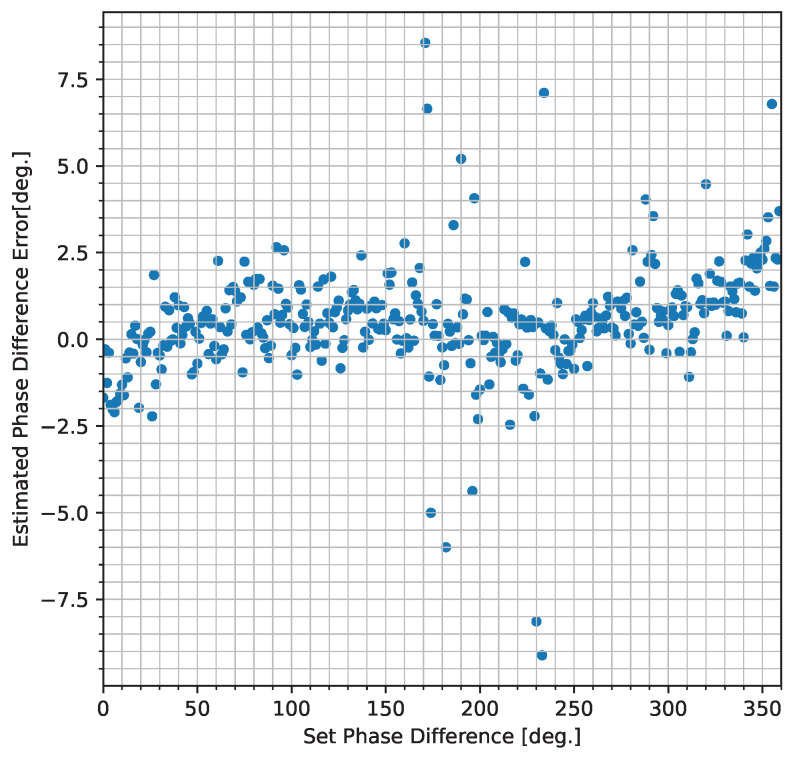
Result of phase difference estimation using the APD.

Figure 8 shows the results of phase difference estimation. The horizontal axis of the graph represents the set phase difference, and the vertical axis represents the estimated phase difference error. The results shows that the estimated phase difference is generally estimated correctly for most of the set phase differences. Additionally, as indicators of accuracy and precision, the mean absolute error (MAE) and standard deviation were calculated, resulting in 1.09° (equivalent to 0.12 m in depth) and 1.65° (equivalent to 0.18 m in depth), respectively.

In addition, 100 measurements for a set phase difference (0° and 90°) were made under the same conditions as in the previous paragraph. The experimental results are shown in Table 2, where the accuracy and precision of the phase difference estimation were approximately 0.75° and 1.05°, respectively.

### 4.2. Phase Difference Measurement Using a CMOS Image Sensor

The experiments in Section 4.1 successfully demonstrated phase difference estimation using a single-pixel APD. Standard CMOS sensors, however, utilize photodiodes that exhibit lower sensitivity than APDs. The realization of ToF imaging with standard CMOS sensors requires both successful phase difference estimation with these less sensitive photodiodes and spatially uniform phase estimation across the sensor. To validate these capabilities, we performed phase difference estimation experiments using a camera equipped with a standard CMOS sensor.

**Figure 9:**
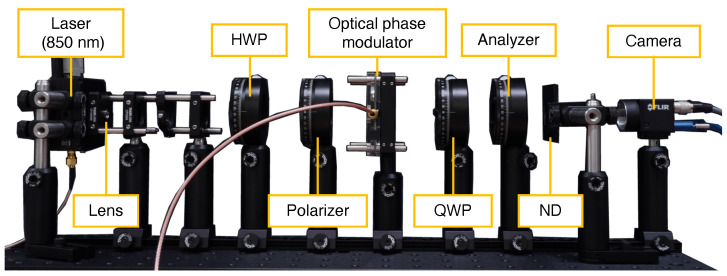
Through-beam type experimental setup with a camera.

The experimental configuration is illustrated in Figure 9. An achromatic lens (C280TMD-B, Thorlabs, Inc., Newton, NJ, USA) was used to collimate the laser beam to a diameter of approximately 6.5 mm. Light intensity was controlled using a half-wave plate to rotate the polarization angle of the polarized laser output. All optical components positioned after the polarizer were consistent with the APD-based experimental setup. We utilized a monochrome camera (BFS-U3-51S5M-C, Teledyne FLIR LLC., Wilsonville, OR, USA) equipped with a standard five-megapixel CMOS sensor (IMX250, Sony Semiconductor Solutions Corporation, Atsugi, Japan), operated without a camera lens. Light intensity was adjusted using a neutral density (ND) filter (NE230B, Thorlabs, Inc., Newton, NJ, USA) positioned before the camera.

Both the light source and polarization modulation frequencies were set to 3.7782 MHz, and the phase difference between their respective signals was controlled via a function generator. Phase estimation was performed using four phase-shifted images acquired at 400 μs exposure time for each set phase difference.

**Figure 10:**
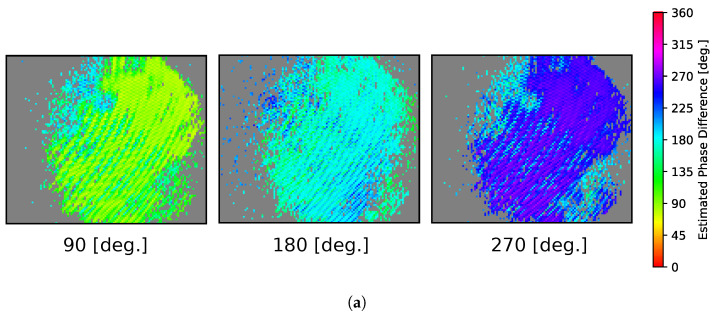

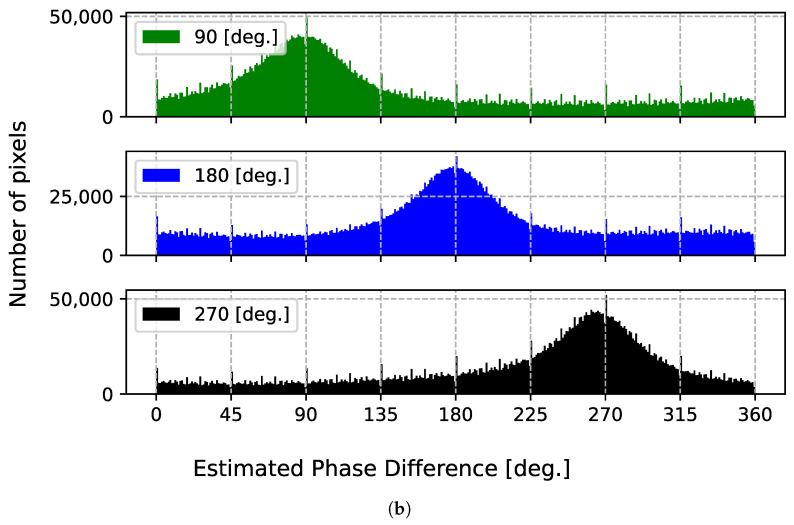
Experimental results of phase difference estimation using the CMOS imaging sensor. (**a**) Estimated phase difference images (90°, 180°, and 270° from left). (**b**) Histograms of estimated phase differences (90°, 180°, and 270° from top).

Figure 10a shows the estimated phase difference images, resulting from left to right at 90°, 180°, and 270°. The estimated phase difference was not uniform across the device plane; phase differences close to the theoretical values were estimated near the center, while different phase values were obtained in the peripheral regions. The histograms of the estimated phase differences (Figure 10b) demonstrate that the peaks of the distributions align with their respective set phases, confirming that accurate phase difference estimation is generally achievable using a multi-pixel camera.

## 5. Evaluation

### 5.1. Depth Imaging with a CMOS Image Sensor

**Figure 11:**
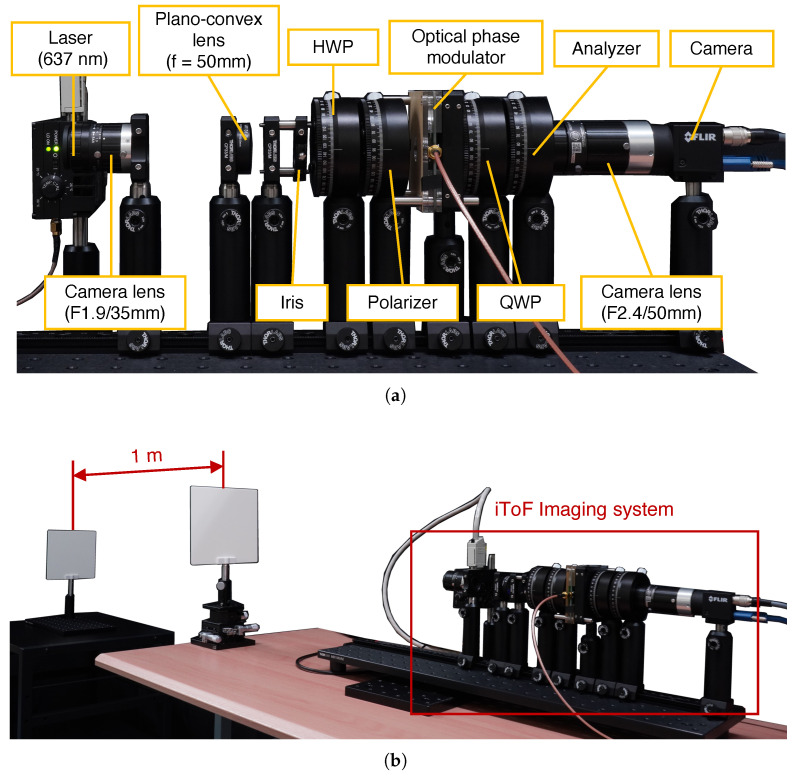
Experimental setup for iToF imaging with a standard CMOS sensor. (**a**) The iToF imaging system. (**b**) Overall view of the setup, including the target object.

First, we evaluate the performance of the proposed system with a standard CMOS image sensor in a typical depth imaging configuration. Figure 11a shows the experimental iToF imaging system setup. A laser at 637 nm wavelength (HL63142DG, Thorlabs, Inc., Newton, NJ, USA) is positioned near an objective lens (F1.9/35 mm). The image formed by the objective lens is relayed using a plano-convex lens (f = 50 mm). Based on experimental results from Section 4.2, the optical phase modulator produced accurate phase estimates near its center but exhibited different phase estimates toward the periphery. Therefore, to ensure parallel light passed only through the center of the optical phase modulator, we employed a plano-convex lens and an iris. Additionally, since the polarization state of reflected light could reduce light intensity by the analyzer, we positioned a half-wave plate (HWP) at an appropriate angle to maximize light transmission. The polarizer, optical phase modulator, QWP, and analyzer were identical to those used in previous experiments. A monochrome camera (BFS-U3-51S5M-C, Teledyne FLIR LLC., Wilsonville, OR, USA) equipped with a standard CMOS sensor (IMX250, Sony Semiconductor Solutions Corporation, Atsugi, Japan) and camera lens (F2.4/50 mm) was positioned after the analyzer, enabling the acquisition of intensity images as a result of optical correlation computations.

Two white boards (EDU-VS1/M, Thorlabs, Inc., Newton, NJ, USA) were positioned as target objects at different depths, with a separation distance of approximately 1 m between them (Figure 11b). The modulation frequency of both the light source and modulator was set to 3.7782 MHz, resulting in a theoretical phase difference of approximately 9.1° between the two board regions. Due to temporal intensity variations, which will be discussed in detail later, we configured the camera with an exposure time of 400 ms and a frame rate of 2 Hz and image acquisition was performed over a 60 s period. A median image was generated from the captured sequence to mitigate the effects of temporal intensity fluctuations. Furthermore, the reflective configuration intensified speckle noise effects, and the speckle patterns varied with changes in the phase difference between the light source and modulator. To minimize these speckle effects, phase difference estimation was performed using images processed with a combination of median and Gaussian filters.

**Figure 12:**
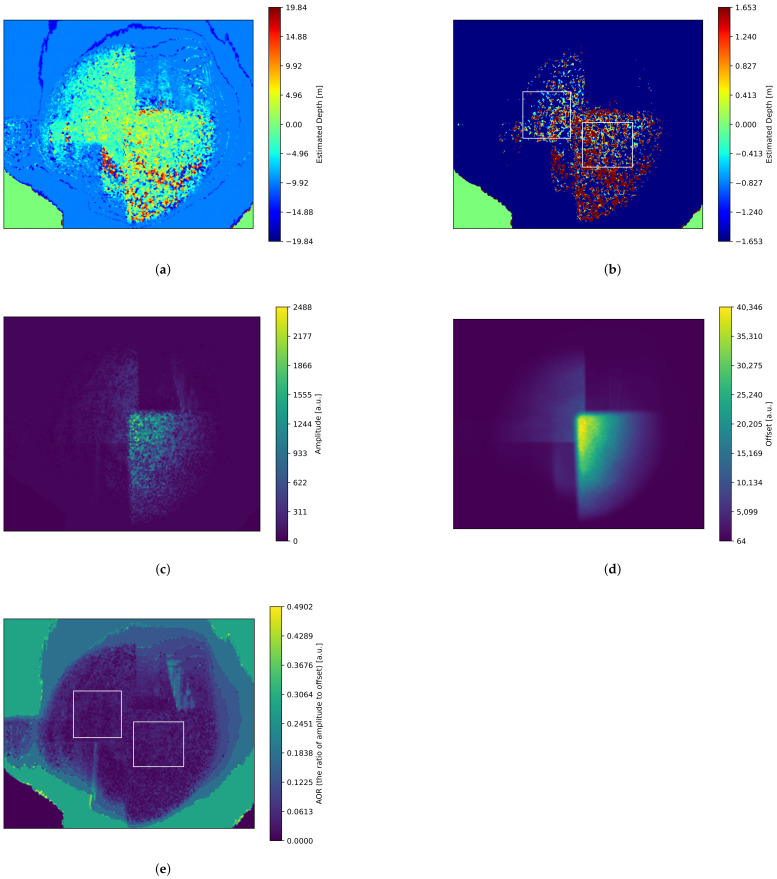
Experimental results of iToF imaging using a standard CMOS sensor. (**a**) Estimated depth image. (**b**) Depth values from (**a**) rescaled from −19.84 m to 19.84 m range to −1.653 m to −1.653 m range. (**c**) Estimated amplitude image. (**d**) Estimated offset image. (**e**) SNR (the ratio of amplitude to offset) image. The white frames in (**b**) and (**e**) indicate the regions of interest (ROIs) in both the front (right box) and rear (left box) board regions.

The experimental results are shown in Figure 12. The estimated depth image (Figure 12a) shows that the depth difference between the two board regions is small. When the depth display range was narrowed to −1.653 m to 1.653 m, subtle differences between the front and rear boards became apparent (Figure 12b). Consequently, we defined regions of interest (ROIs) for the four phase-shifted images in both the front and rear board regions (within the white frames shown in Figure 12b) and estimated the phase using the median values within these ROIs. As a result, the estimated depth in the front board region was −1.07 m, while in the rear board region, it was −2.03 m, yielding a depth difference of 0.96 m. This indicates that the depth was estimated approximately correctly.

Figure 12c,d represent the estimated amplitude and offset images, respectively, while Figure 12e is the amplitude-to-offset ratio (AOR) image computed from them. The AOR is significantly low, with the mean and standard deviation within the white frames (shown in Figure 12e) being 0.031 and 0.014 for the front side and 0.029 and 0.012 for the rear side. Since the sensor’s dynamic range is limited, a low AOR reduces the effective range of amplitude, increasing quantization errors. The primary cause of the low AOR is the small angular change in linearly polarized light due to polarization modulation, which results from insufficient power supplied to the optical phase modulator. By inputting a properly powered sinusoidal signal into the optical phase modulator, the amplitude of the polarization-modulated light transmission can approach its ideal value, leading to improvements in both AOR and depth estimation accuracy.

Additionally, the amplitude image exhibits a speckled pattern caused by laser speckle. Speckle arises from interference in the wavefront scattered by a rough surface, leading to large variations between bright and dark points. When attempting to capture an overall bright image, the bright speckle points tend to saturate easily. Once brightness saturation occurs, phase calculation based on the brightness differences ratio becomes impossible, making speckle noise reduction necessary. One possible approach is to place a rotating diffuser in front of the light source.

Furthermore, as mentioned earlier, temporal brightness fluctuations unrelated to polarization modulation were observed. These fluctuations were not only larger than the brightness variations induced by phase shifts in polarization modulation but also made it difficult to capture the four phase-shifted images within a short period. As a result, the four-phase images were significantly affected by these fluctuations, leading to incorrect phase difference estimation. To mitigate this effect, we generated a median image from a large set of images acquired over 60 s, effectively reducing the impact of temporal variations. Although the exact cause of these fluctuations has not yet been identified, one possible factor is the instability of the laser source output due to thermal effects. In this case, using a VCSEL, which offers greater thermal stability than conventional lasers, could resolve this issue. Additionally, if the previously mentioned issue of small polarization modulation amplitude is resolved, and the brightness variations induced by the correlation between light source modulation and polarization modulation become sufficiently large, this problem can also be mitigated.

### 5.2. Depth Imaging with a Polarization Image Sensor

Second, we evaluate an extended system with a polarization image sensor. As described in Section 2.2, the four phase shifts required for phase estimation can be achieved by varying the analyzer angle through 0°, 45°, 90°, and 135°. Thus, using a polarization image sensor enables the acquisition of four phase-shifted images in a single capture. However, when the angular variation of linear polarization due to polarization modulation is small, the intensity varies significantly depending on both the offset angle of polarization modulation and the analyzer angle. Since this intensity variation is not caused by the correlation computation from polarization modulation, the phase difference cannot be estimated from the four polarization angle images. Meanwhile, the phase shift magnitude of polarization modulation can also be set through the phase of the signal applied to the optical phase modulator. Therefore, by combining phase shift settings through electrical signals with phase shifts from analyzer angles, we can suppress intensity variations caused by the polarization modulation offset angle and analyzer angle. Since the polarization image sensor is equipped with detectors at different angles, the phase difference can be estimated with only two measurements.

The experimental setup is modified from the standard CMOS sensor configuration shown in Figure 11 by removing the analyzer and replacing the camera with a polarization camera (BFS-U3-51S5P-C, Teledyne FLIR LLC., Wilsonville, OR, USA). The target objects remained identical, with the distance between the two white boards maintained at 1 m. The phases of signals applied to the optical phase modulator are set to 0° and 180°, while analyzer angles of 0° and 135° are employed. The combinations of optical phase modulator signal phases and analyzer angles used to acquire the phase-shifted images are shown in Table 3. As with the standard camera setup, to minimize the effects of temporal intensity variations, the camera exposure time was set to 100 ms with a frame rate of 2 Hz, and images were captured over a 60 s period. The median images computed from these captured sequences were used for phase estimation calculations.

**Figure 13:**
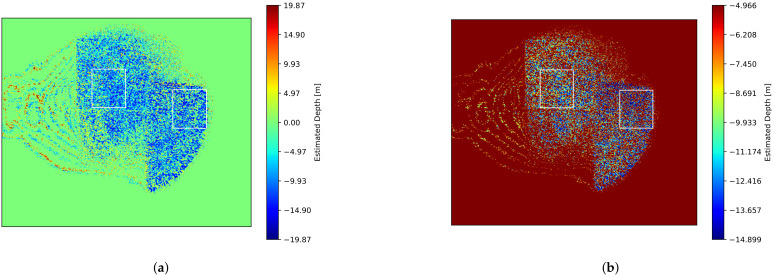

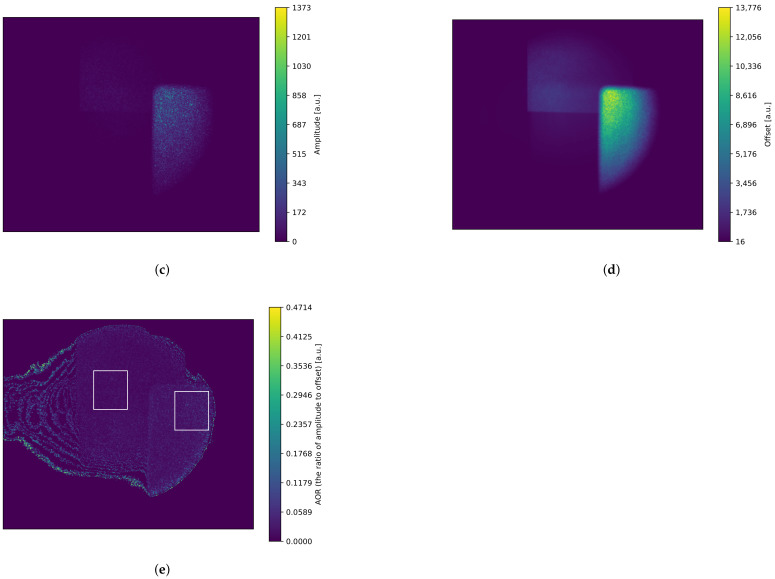
Experimental results of iToF imaging using a polarization CMOS sensor. (**a**) Estimated depth image. (**b**) Depth values from (**a**) rescaled from −19.87 m to 19.87 m range to −14.899 m to −4.966 m range. (**c**) Estimated amplitude image. (**d**) Estimated offset image. (**e**) SNR (the ratio of amplitude to offset) image. The white frames in (**b**) and (**e**) indicate the regions of interest (ROIs) in both the front (right box) and rear (left box) board regions.

The experimental results are presented in Figure 13. Figure 13a shows the estimated depth map, where little depth difference is observed between the two plate regions. When the depth display range was constrained to −14.899 m to −4.966 m, a difference between the front and rear boards became visible (Figure 13b). As in previous experiments, ROIs were established for the four phase-shifted images in both front and rear board regions (indicated by white frames in Figure 13b). Depth estimation using the median values within these ROIs yielded a depth of −11.2 m for the front board region and −10 m degrees for the rear board region, resulting in a depth difference of 1.2 m. While this shows a slightly larger depth estimation error compared to the standard CMOS sensor experiment (which required four measurements with fixed analyzer angles and varying optical phase modulator input phases), the use of a polarization camera successfully reduced the number of required measurements to two.

Figure 13c,d represent the estimated amplitude and offset images, respectively, while Figure 13e is the AOR image computed from them. The AOR is significantly low, with the mean and standard deviation within the white frame (shown in Figure 13e) being 0.019 and 0.035 for the front side and 0.019 and 0.012 for the rear side. It is even lower compared to the case with a monochrome camera. As explained in Section 3.3, when the polarization modulation amplitude is small, an appropriate angle setting becomes crucial. However, since the polarization camera has a fixed analyzer angle, the AOR was further reduced. If the AOR is improved, it will be possible to acquire the phase-shifted images required for the four-bucket method in a single exposure.

## 6. Conclusions

In this paper, we proposed a novel polarization-modulated optical homodyne system for iToF imaging using standard CMOS sensors. The system employs a resonant photoelastic modulator combined with polarization optics to enable optical correlation for depth measurement, achieving high spatial resolution without specialized ToF sensor arrays. Through experimental validation, the system successfully demonstrated depth measurement capabilities using both standard CMOS and polarization image sensors, with the latter reducing required measurements from four to two shots. The experimental results show accurate depth estimation between two target boards separated by 1 m, achieving measurements of 0.96 m and 1.2 m using standard and polarization CMOS sensors, respectively.

Currently, due to insufficient driving power supplied to the modulator, the polarization modulation amplitude is reduced, necessitating analyzer angle adjustments and time-averaging processing. Additionally, despite employing the high-precision phase detection method of the homodyne approach, the depth estimation accuracy remains comparable to that of the heterodyne method [18,20]. If the modulator is driven with sufficient power and the polarization modulation amplitude becomes sufficiently large, these issues will be resolved, enabling high-precision depth measurement in a single acquisition, which is a key advantage of the homodyne method.

Additionally, the resonant photoelastic modulator has advantages such as being thin and capable of modulation over a large area. However, it has a limitation where the modulation frequency is determined by the substrate thickness. The device fabricated in this study had a thickness of 0.5 mm, resulting in a modulation frequency of approximately 3.7 MHz. Since higher modulation frequencies lead to higher depth estimation resolution, the depth resolution of this method remains low. Increasing the modulation frequency requires a thinner substrate, but this, in turn, demands higher driving power.

While an electro-optic modulator can achieve extremely high-speed polarization modulation, its small aperture size limits its applicability to multi-pixel imaging [12]. Additionally, while image intensifiers [10,11] and electro-absorption modulators [13,14,15,16] can achieve modulation over a large area at frequencies comparable to conventional ToF cameras, they do not rely on polarization modulation. As a result, even when combined with a polarization camera, they do not enable a reduction in the number of measurements. As different types of modulators each have their own advantages and limitations, a polarization modulator capable of high-speed, large-area modulation remains essential for methods utilizing a high-speed optical shutter and a standard CMOS sensor.

Despite these current limitations, the research demonstrates the potential for achieving high-resolution depth imaging using conventional image sensors through polarization-based optical correlation techniques. The approach of enhancing the spatial resolution of iToF cameras through optical correlation operation, as demonstrated in this study, is inherently valuable. Moving forward, the development of modulation devices for ToF imaging is expected to continue, contributing to the advancement of this field.

## Figures and Tables

**Table 1:** Comparison table of methods.

Method	Correlation	Phase Detection	Number of Pixels	Number of Measurements	Depth Resolution
Conventional iToF	Electrical	Homodyne	Low	2 to 4	Middle
SR [4,5,6]	Electrical	Homodyne	Middle	2 [6], Multiple	Middle
SWI [7,8,9]	Optical	Heterodyne	**High**	**1 [9]**, Multiple	**High**
HOS-II [10,11]	Optical	Heterodyne	**High**	Multiple	Middle
HOS-EOM [12]	Optical	Homodyne	Single (scanning)	4×2 (2 frequencies)	**High**
HOS-EAM [13,14,15,16]	Optical	Heterodyne	**High**	4	Middle
HOS-PEM [17,18,19,20,21,22]	Optical	Heterodyne	**High**	Multiple	Low
HOS-PEM (Ours)	Optical	Homodyne	**High**	4, **1 (Pol-Cam)**	Low

Abbreviations: SR, super-resolution; SWI, synthetic wavelength imaging; HOS, highspeed optical shutter; II, image intensifier; EOM, electro-optic modulator; EAM, electro-absorption modulator; PEM, photo-elastic modulator; Pol-Cam, polarization camera. Bold values indicate superior performance in each respective category.

**Table 2:** The result of 100 repeated measurements with a fixed set phase difference. The values in parentheses represent the phase difference converted into virtual depth.

Set Phase Difference	Mean Absolute Error (MAE)	Standard Deviation
0° ( 0 m)	0.66° ( 0.073 m)	1.1° ( 0.12 m)
90° ( 9.9 m)	0.84° ( 0.093 m)	1.0° ( 0.11 m)

**Table 3:** Combined setting of phase of optical phase modulator signal and detector angle for 2-shot phase estimation using polarization camera.

Four Phase Shift	Optical Phase Modulator Signal Phase	Analyzer Angle
0°	0°	0°
90°	180°	135°
180°	180°	0°
270°	0°	135°

## Data Availability

The original contributions presented in this study are included in the article. Further inquiries can be directed to the corresponding authors.
